# Proteus mirabilis Employs a Contact-Dependent Killing System against Competing *Enterobacteriaceae*

**DOI:** 10.1128/mSphere.00321-21

**Published:** 2021-07-28

**Authors:** Dara Kiani, William Santus, Kaitlyn A. Kiernan, Judith Behnsen

**Affiliations:** a Department of Microbiology and Immunology, University of Illinois at Chicago, College of Medicine, Chicago, Illinois, USA; University of Maryland Medical Center

**Keywords:** *Proteus mirabilis*, interbacterial competition, bacterial killing, *Enterobacteriaceae*

## Abstract

Many bacterial species employ systems for interference competition with other microorganisms. Some systems are effective without contact (e.g., through secretion of toxins), while other systems (e.g., type VI secretion system [T6SS]) require direct contact between cells. Here, we provide the initial characterization of a novel contact-dependent competition system for Proteus mirabilis. In neonatal mice, a commensal P. mirabilis strain apparently eliminated commensal Escherichia coli. We replicated the phenotype *in vitro* and showed that P. mirabilis efficiently reduced the viability of several *Enterobacteriaceae* species but not Gram-positive species or yeast cells. Importantly, P. mirabilis strains isolated from humans also killed E. coli. A reduction of viability occurred from early stationary phase to 24 h of culture and was observed in shaking liquid media as well as on solid media. Killing required contact but was independent of T6SS, which is the only contact-dependent killing system described for P. mirabilis. Expression of the killing system was regulated by osmolarity and components secreted into the supernatant. Stationary-phase P. mirabilis culture supernatant itself did not kill but was sufficient to induce killing in an exponentially growing coculture. In contrast, killing was largely prevented in media with low osmolarity. In summary, we provide the initial characterization of a potentially novel interbacterial competition system used by P. mirabilis.

**IMPORTANCE** The study of bacterial competition systems has received significant attention in recent years. These systems are important in a multitude of polymicrobial environments and collectively shape the composition of complex ecosystems like the mammalian gut. They are also being explored as narrow-spectrum alternatives to specifically eliminate problematic pathogenic species. However, only a small fraction of the estimated number of interbacterial competition systems has been identified. We discovered a competition system that is novel for Proteus mirabilis. Inspired by an observation in infant mice, we confirmed *in vitro* that P. mirabilis was able to efficiently kill several *Enterobacteriaceae* species. This killing system might represent a new function of a known competition system or even a novel system, as the observed characteristics do not fit with described contact-dependent competition systems. Further characterization of this system might help understand how P. mirabilis competes with other *Enterobacteriaceae* in various niches.

## INTRODUCTION

Bacteria frequently inhabit densely populated environments like the soil or the human gastrointestinal tract. In these environments, competitive interactions, such as interference and exploitative competition, are common (1). In exploitative competition, bacteria compete for common nutrient sources, whereas in interference competition bacteria employ systems that directly affect communication or viability of competitor bacteria. A multitude of different competition systems have been described and are continuing to be discovered (2, 3). They are used for “bacterial warfare” in the gastrointestinal tract by commensal and pathogenic species and are thought to collectively shape the composition of the gut microbiota (4, 5). In the current antibiotic crisis, tools to precision edit the microbiota and to eliminate only problematic species instead of the majority of gut bacteria are highly desirable (1, 6, 7). Narrow-spectrum competition mechanisms employed by commensals to kill closely related species have therefore received significant attention in recent years (8–12).

Bacterial competition systems are classified as either contact dependent or contact independent. Contact-independent competition is mediated through secreted compounds like classical antibiotics, bacteriolytic enzymes, or bacteriocins, colicins, and microcins (13). Contact-dependent mechanisms have been discovered predominantly during the last decade and include, among others, type VI secretion system (T6SS) (14–17) and type VII secretion system (T7SS) (18)-mediated effector translocation, contact-dependent growth inhibition (CDI) (19–23), contact-dependent inhibition by glycine zipper proteins (Cdz) (24), and microcin proximity-dependent inhibition (MccPDI) (25). These systems require direct contact between cells, as effector molecules do not diffuse from the producing cell but are transferred when cells touch, e.g., through the molecular syringe complex of the T6SS. Functions of the effector molecules are wide ranging and include interference with protein synthesis and induction of pore formation in the bacterial membrane of the target cell (13).

*Enterobacteriaceae* members are successful colonizers of the mammalian gut, in the form of commensals, pathobionts, or pathogens. One of the most frequent *Enterobacteriaceae* species in the mammalian gut is Escherichia coli. However, species from other genera, including Klebsiella, Enterobacter, *Serratia*, and Proteus, can be frequently isolated from both healthy infants and adults (26, 27). In an undisturbed adult gut environment, levels of *Enterobacteriaceae* are generally low (28, 29). However, they dominate the microbiota in the following two instances: in the infant gut (30, 31) or during dysbiosis of the adult gut (32). Blooms of commensal *Enterobacteriaceae* are often associated with disease, e.g., inflammatory bowel disease (IBD) or necrotizing enterocolitis in infants (NEC) (33, 34). However, probiotic species of *Enterobacteriaceae* also exist. The probiotic E. coli Nissle 1917 is used to treat intestinal diseases like diarrhea in infants (35). Our study focuses on E. coli and Proteus mirabilis. P. mirabilis is a known pathogen of the urogenital tract but is also frequently present as a commensal in the gastrointestinal tract. However, due to sampling methods, Proteus species abundance is often underestimated. Proteus species were found in only 7.8% of healthy adult fecal samples (36) but are present in 46% of jejunal and duodenal mucus samples (37). They are more frequently isolated from patients with diarrhea and Crohn’s disease, but a direct role in the disease has not yet been established (38). Proteus species and E. coli were isolated from the majority of cesarean-born infants in Pakistan (39) indicating that P. mirabilis and E. coli are both found in the human gut. Recently, the genus Proteus has been reclassified to the new family *Morganellaceae*, forming together with *Enterobacteriaceae* the order *Enterobacteriales* (40). For simplicity, we use the old terminology throughout the manuscript.

Here, we characterize a contact-dependent competition system employed by P. mirabilis to compete with other members of the *Enterobacteriaceae* family. We initially observed the competition between P. mirabilis and E. coli *in vivo* in mouse pups and replicated the phenotype *in vitro*. The killing system functions independently of T6SS, the only contact-dependent killing system described for P. mirabilis. The system is therefore novel for P. mirabilis and might represent a novel system for *Enterobacteriaceae* in general. The focus and scope of the manuscript is the initial characterization of this P. mirabilis competition system *in vitro.* Further studies are warranted to reveal the genetic components, regulatory circuits, and *in vivo* importance of this competition system, as well as its prevalence in other species.

## RESULTS

**FIG 1:**
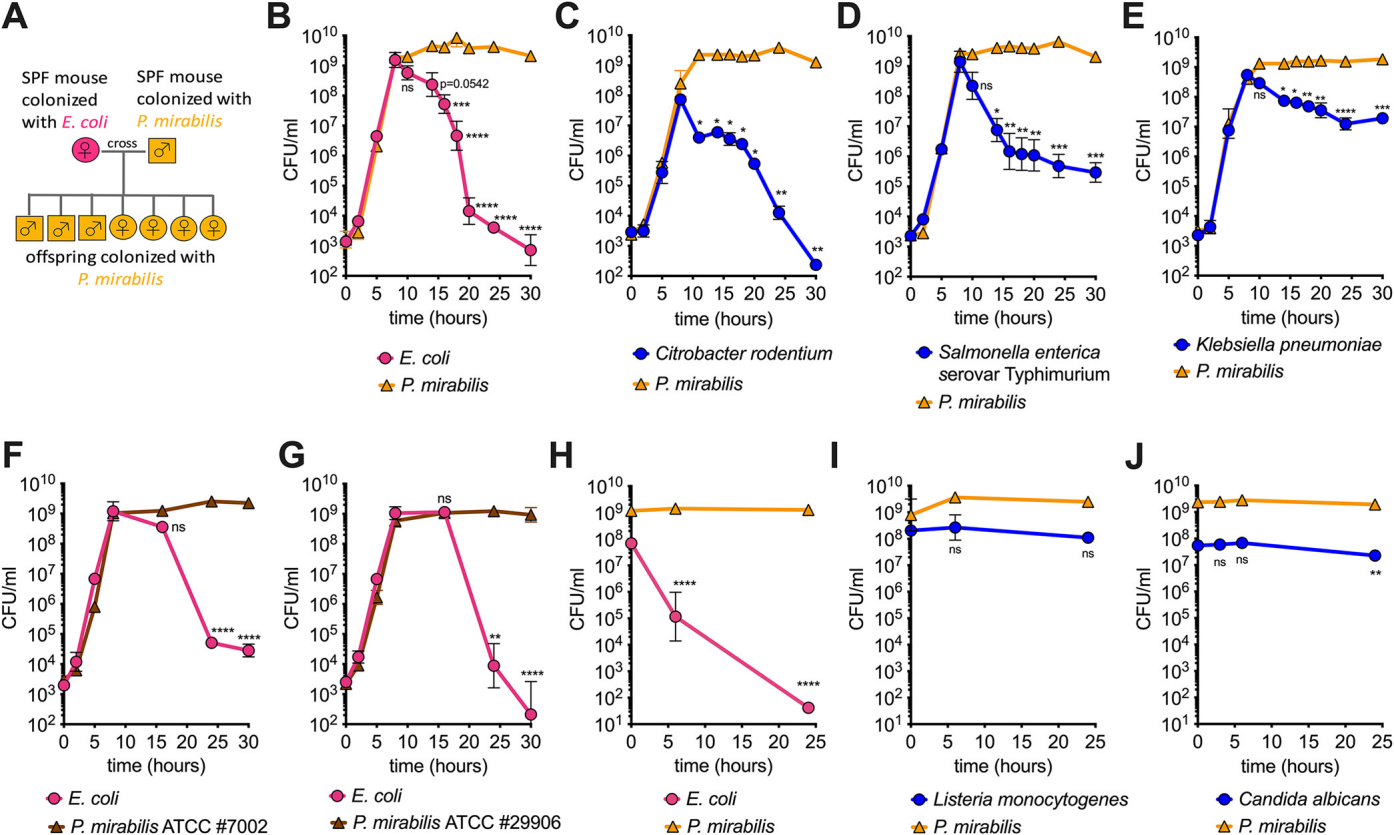
Proteus mirabilis reduces the viability of *Enterobacteriaceae*. (A) Schematic representation of breeding scheme. The offspring of an SPF female mouse colonized with Escherichia coli and an SPF male mouse colonized with P. mirabilis were exclusively colonized with P. mirabilis. (B to E) Shaking liquid growth curve of P. mirabilis isolated from mice and different *Enterobacteriaceae* species in coculture in LB, as follows: E. coli (B), Citrobacter rodentium (C), Salmonella enterica serovar Typhimurium (D), and Klebsiella pneumoniae (E). (F and G) Shaking liquid growth curve of P. mirabilis ATCC 7002 (F) or ATCC 29906 (G) and E. coli in coculture in LB. (H to J) “Killing assay” with P. mirabilis and target species E. coli (H), Gram-positive Listeria monocytogenes (I), or yeast Candida albicans (J). The P. mirabilis to target species ratio was ∼10:1. All data represent mean ± SEM of at least three biological replicates. If error bars are not visible, the error is smaller than the symbol size. For B through G, a one-way ANOVA was performed comparing the 8-hour time point (upon entry to stationary phase) with the remaining time points (stationary phase). For H through J, a one-way ANOVA was performed comparing the 0-hour time point and remaining time points. *, *P* < 0.05; **, *P* < 0.01; ***, *P* < 0.001; ****, *P* < 0.0001; ns, not significant.

In any natural environment where bacteria reside, such as the gastrointestinal tract or aquatic or soil environments, bacteria will compete with one another in order to survive and replicate. We observed what appeared to be efficient competition between two commensal strains of *Enterobacteriaceae* in mice. A female specific-pathogen-free (SPF) mouse naturally colonized with Escherichia coli was mated with a male SPF mouse naturally colonized with Proteus mirabilis. Surprisingly, pups were exclusively colonized with P. mirabilis, and no E. coli was identified in their feces postweaning (Fig. 1A). We expected E. coli and P. mirabilis to be transferred to pups through intimate contact and coprophagy. As we recovered only P. mirabilis from the pups, we investigated this apparent competitive advantage of P. mirabilis.

### P. mirabilis reduces the viability of *Enterobacteriaceae* species.

An analysis of different lines of mice revealed that adult mice were either colonized with E. coli (41) or P. mirabilis but not both species at the same time (see Fig. S1A in the supplemental material). Strains also did not transfer during cohousing of adult mice (Fig. S1B). We isolated E. coli and P. mirabilis and grew the two strains in a coculture in lysogeny broth (LB). During the exponential phase of growth, both strains grew equally well. In stationary phase, P. mirabilis maintained its viability. The number of viable E. coli on the other hand declined quickly, reaching a rate of 1,600-fold loss of viable cells within 2 hours (Fig. 1B). In a monoculture of E. coli, we observed no loss of viability in stationary phase (Fig. S1C). P. mirabilis also significantly reduced the viability of Citrobacter rodentium, a rodent pathogen and model organism for enterohemorrhagic Escherichia coli (EHEC) in mice (Fig. 1C), and the human pathogen Salmonella enterica serovar Typhimurium (Fig. 1D). In contrast, the viability of another *Enterobacteriaceae* member, Klebsiella pneumoniae, was not reduced to the same extent as the others (Fig. 1E). The phenotype was independent of capsule, as no increase in susceptibility was seen at 24 hours with a capsule-deficient K. pneumoniae strain (Fig. S1D). Importantly, the ability to kill extends to other P. mirabilis strains. Two isolates of P. mirabilis (the type strain and a human isolate) also killed E. coli in shaking liquid media (Fig. 1F and G). We next tested if P. mirabilis can reduce the viability of the Gram-positive bacterium Listeria monocytogenes and the eukaryotic yeast Candida albicans. Since both species grow poorly in LB, we directly assessed viability by adding prey species to stationary cultures of P. mirabilis. P. mirabilis drastically reduced the viability of E. coli in this assay (Fig. 1H), but we observed no change in the viability of L. monocytogenes (Fig. 1I) or C. albicans (Fig. 1J).

10.1128/mSphere.00321-21.1 FIG S1 Colonization status of mice and *in vitro Enterobacteriaceae* growth and competition. (A) E. coli and P. mirabilis colonization status of 78 C57BL/6 mice from different cages and mouse lines at a UCI vivarium. Grey box, average baseline *Enterobacteriaceae* colonization. (B) C57BL/6 mice of the same line colonized with either P. mirabilis or E. coli cohoused for 276 (cage 1) or 581 days (cage 2). Pink symbol, E. coli CFU; yellow symbol, P. mirabilis CFU. (C) E. coli cells were inoculated in LB, and viability was measured over a 24-hour period. (D) Viability in a 24-h coculture of P. mirabilis isolated from mice and Klebsiella pneumoniae WT or capsule-deficient mutant in LB. (C and D) All data represent mean ± SEM of at least three biological replicates. If error bars are not visible, the error is smaller than the symbol size. LOD, limit of detection; ns, not significant. Download FIG S1, JPG file, 0.5 MB. Copyright © 2021 Kiani et al. 2021 Kiani et al. https://creativecommons.org/licenses/by/4.0/ This content is distributed under the terms of the Creative Commons Attribution 4.0 International license.

### Killing of E. coli is an active process that requires direct contact and live P. mirabilis cells.

**FIG 2:**
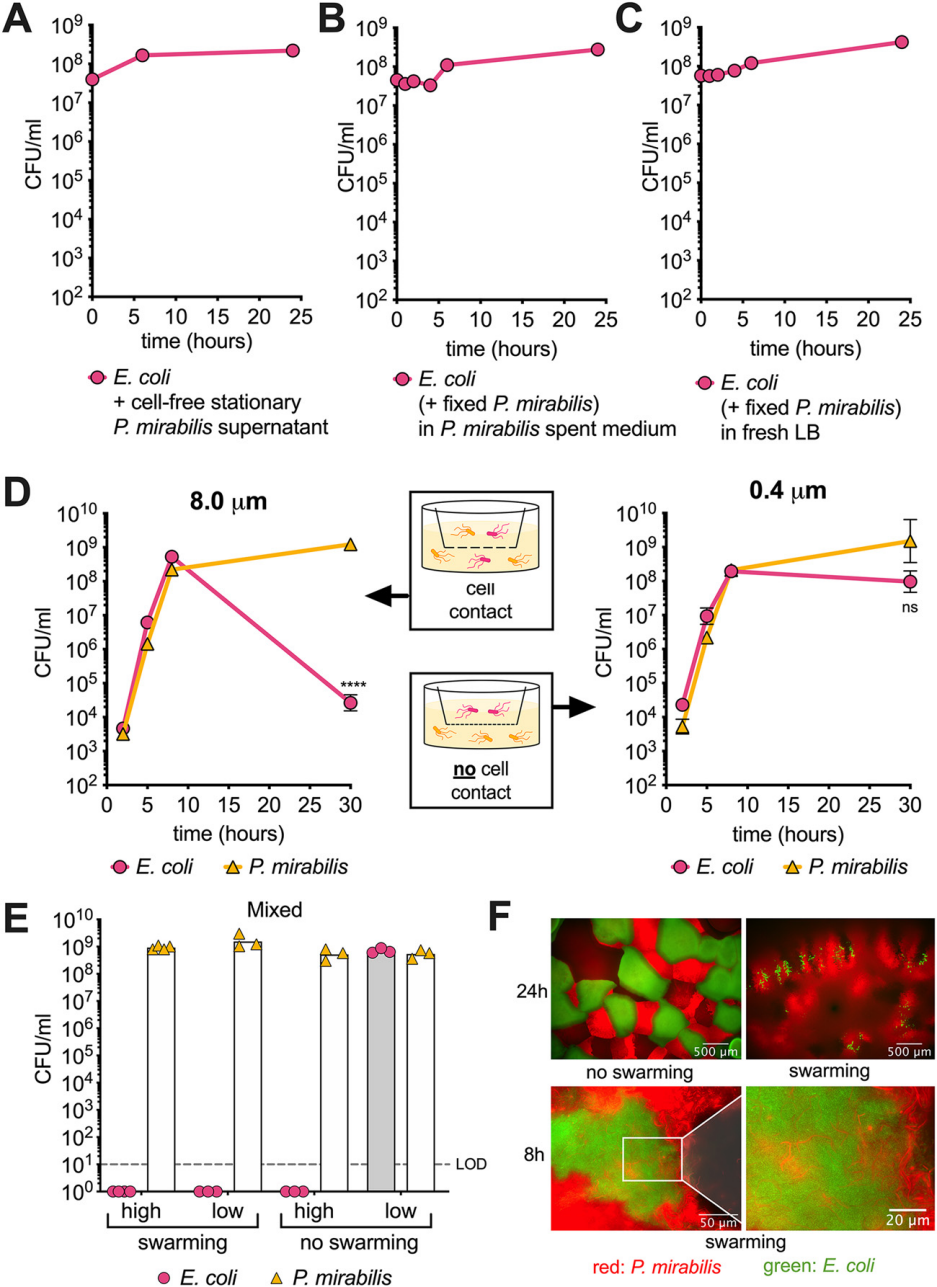
Contact and live cells are required for P. mirabilis to kill E. coli. (A to C) E. coli cells grown to stationary phase for 16 hours were added to cell-free sterile-filtered supernatant of P. mirabilis (A), stationary-phase P. mirabilis supernatant and formalin-fixed P. mirabilis cells (B), or fresh LB and formalin-fixed P. mirabilis cells (C). (D) Cultures of P. mirabilis and E. coli cells in 6-well plates separated by membranes with 8.0-μm or 0.4-μm pore sizes. (E) Solid surface assay with mixed cultures of P. mirabilis and E. coli on swarming permissive (LB) agar or nonswarming permissive (MacConkey) agar in high density (10^8^ CFU of each strain) or low density (10^3^ CFU of each strain) of cells in each spot. All data represent mean of at least three biological replicates. (F) Top: fluorescent P. mirabilis (red) and E. coli (green) seeded in low density on swarming permissive or nonswarming permissive media and imaged after 24 h. Bottom: imaging after 8 h on swarming permissive agar; right panel represents magnification of inset. For D, a Welch’s *t* test was performed between the 8-hour time point and the 30-hour time point. ****, *P* < 0.0001; ns, not significant; LOD, limit of detection. If error bars are not visible, the error is smaller than the symbol size.

We next wanted to test if P. mirabilis killing requires contact or if it occurs via the release of proteins, molecules, membrane vesicles, or phages into the surrounding medium. We found that in the cell-free stationary supernatant of P. mirabilis (pH ∼7.6), E. coli cells remained viable (Fig. 2A). P. mirabilis therefore does not seem to reduce the viability of E. coli through a contact-independent mechanism. Formalin-fixed stationary P. mirabilis cells also failed to reduce the viability of E. coli in P. mirabilis stationary-phase supernatant (Fig. 2B) or in fresh LB (Fig. 2C). Killing therefore requires live P. mirabilis cells.

To understand whether killing requires direct contact, we separated E. coli and P. mirabilis cultures with membranes of different pore sizes (Fig. 2D). An 8.0-μm membrane allows the exchange of the secretome and physical contact between cells. We observed about a 20,000-fold reduction in E. coli viability at 30 hours compared with 8 hours (Fig. 2D, left panel). In contrast, we observed no significant loss in E. coli viability with an 0.4-μm membrane that blocked physical contact, which allowed only the passage of the secretome (Fig. 2D, right panel). P. mirabilis-mediated killing is therefore an active process that requires live P. mirabilis in direct contact with E. coli.

### P. mirabilis kills *Enterobacteriaceae* on a solid surface in a contact-dependent manner.

The contact-dependent weapons of Gram-negative species have predominantly been reported to be active and expressed on solid media, with only very few being active in shaking liquid culture (42, 43). We therefore tested P. mirabilis competition with different *Enterobacteriaceae* species on solid media. We inoculated P. mirabilis and E. coli, *S.* Typhimurium, or K. pneumoniae in either high cell density (10^8^ CFU) or low cell density (10^3^ CFU) on two different media. On LB agar, P. mirabilis can swarm and cover an entire agar plate within 24 h. On MacConkey agar, swarming is inhibited and P. mirabilis forms distinct colonies. We recovered similar numbers from all conditions when only one species was present (see Fig. S2A, C in the supplemental material). When we cultured P. mirabilis and E. coli together on nonswarming permissive MacConkey agar, killing of E. coli was dependent on seeding density. When P. mirabilis and E. coli cells were seeded at a high density, they came in direct contact with one another, as confirmed by fluorescence microscopy (Fig. S2F). In this scenario, we recovered no viable E. coli cells at 24 h. When cells were seeded at low density and thus were physically separated, we recovered equal numbers of E. coli and P. mirabilis cells regardless of incubation temperature (Fig. 2E and Fig. S2B). At 24 h after inoculation, fluorescence microscopy showed microcolonies of P. mirabilis (red) and E. coli (green) that touched, but no P. mirabilis was found within the boundaries of E. coli colonies (Fig. 2F). Contrary to MacConkey agar, we recovered no viable E. coli cells on LB agar, regardless of the seeding density. Microcolonies of E. coli formed with low seeding density also on LB agar. However, here, P. mirabilis was highly mobile and could be seen actively invading E. coli colonies 8 h postinoculation (Fig. 2F; see Movie S1 in the supplemental material). Consequently, E. coli colonies are largely disrupted 24 h postinoculation and only small pockets of E. coli cells can be found (Fig. 2F, Fig. S2G). Interestingly, *S.* Typhimurium was more susceptible to P. mirabilis-mediated killing on solid surfaces than in liquid culture. With high seeding density, we recovered no viable *S.* Typhimurium cells, irrespective of agar type (Fig. S2D). K. pneumoniae on the other hand showed only reduced viability when P. mirabilis was able to swarm and not on non-swarming permissive MacConkey agar (Fig. S2E).

10.1128/mSphere.00321-21.2 FIG S2 P. mirabilis kills *Enterobacteriaceae* when swarming on solid surfaces. (A to E) Cultures of P. mirabilis and E. coli were inoculated on the solid surface of swarming permissive (LB) agar or nonswarming permissive (MacConkey) agar in high density (10^8^ CFU of each strain) or low density (10^3^ CFU of each strain) of cells in each spot and incubated for 24 h at 30°C. All data represent mean of at least three biological replicates (single and mixed high-density culture of E. coli on MacConkey two biological replicates). LOD, limit of detection. (A) Monocultures of P. mirabilis or E. coli. (B) Mixed cultures of P. mirabilis and E. coli. (C) Monocultures of Klebsiella pneumoniae or Salmonella enterica serovar Typhimurium. (D) Mixed cultures of P. mirabilis and *S*. Typhimurium. (E) Mixed cultures of P. mirabilis and K. pneumoniae. (F) Fluorescence imaging of P. mirabilis (red) and E. coli (green) in low density or high density immediately after seeding (0 h). (G) Fluorescence imaging of P. mirabilis (red) and E. coli (green) on swarming and nonswarming permissive media 24 h after seeding. Download FIG S2, JPG file, 0.8 MB. Copyright © 2021 Kiani et al. 2021 Kiani et al. https://creativecommons.org/licenses/by/4.0/ This content is distributed under the terms of the Creative Commons Attribution 4.0 International license.

10.1128/mSphere.00321-21.6 MOVIE S1 P. mirabilis can penetrate E. coli microcolonies on swarming permissive agar. Time lapse of fluorescence imaging of P. mirabilis (red) and E. coli (green) on fresh LB agar plate. Images were captured every 25 seconds for 30 minutes. Blue arrows indicate P. mirabilis swarmer cells invading the E. coli microcolony. Of note, the agar dries over the course of the time lapse and the reduced water content slows P. mirabilis movement. Scale bar, 50 μm. Download Movie S1, AVI file, 4.1 MB. Copyright © 2021 Kiani et al. 2021 Kiani et al. https://creativecommons.org/licenses/by/4.0/ This content is distributed under the terms of the Creative Commons Attribution 4.0 International license.

### Killing is not mediated through the P. mirabilis type VI secretion system (T6SS).

**FIG 3:**
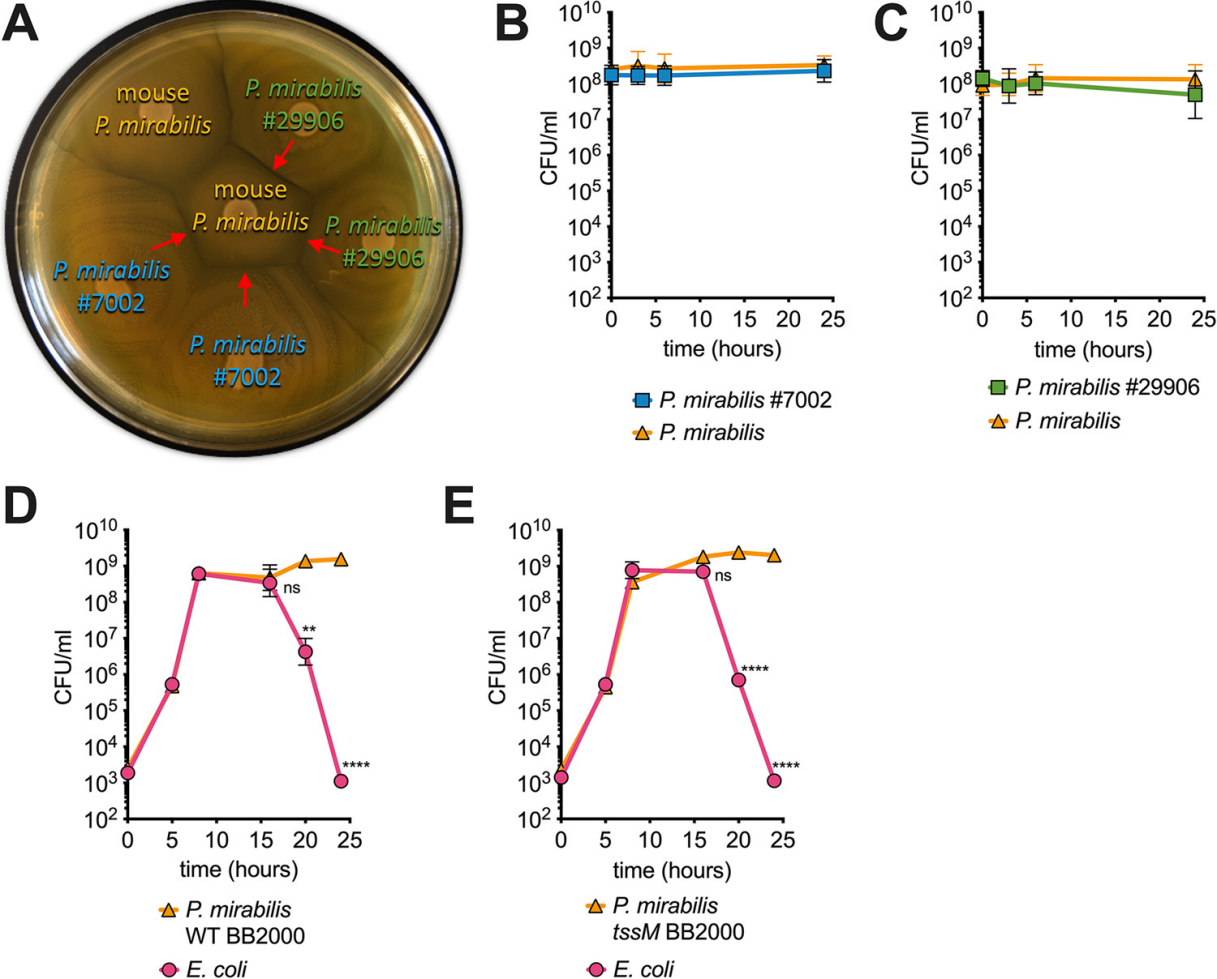
Killing is not mediated through the P. mirabilis T6SS. (A) Representative image (*n* = 3) of mouse P. mirabilis, P. mirabilis ATCC 7002, and P. mirabilis ATCC 29906 that were spotted in duplicate on LB agar and incubated for >24 h. Red arrows indicate strong Dienes line formation. (B and C) Liquid culture “killing assay” of mouse P. mirabilis and P. mirabilis ATCC 7002 (B) or P. mirabilis ATCC 29906 (C). (D and E) Coculture in LB of E. coli and BB2000 WT (D) or BB2000 mutant *tssM* (E), which lacks a functional T6SS. All data represent mean ± SEM of at least three biological replicates. A one-way ANOVA was performed between the 8-hour time point (upon entry to stationary phase) and the remaining time points (stationary phase). **, *P* < 0.01; ****, *P* < 0.0001; ns, not significant.

The T6SS is a well-characterized interbacterial killing system (5, 44–46). On solid surfaces, P. mirabilis uses T6SS against nonkin clonemates (46, 47). However, we observed that P. mirabilis killed antagonists in shaking liquid media (Fig. 1). The current literature does not provide evidence that P. mirabilis or any other organism uses T6SS in shaking liquid media (14, 47–49). Nevertheless, we tested whether P. mirabilis utilizes T6SS to reduce viability of E. coli. One key characteristic of P. mirabilis is its ability to swarm on permissive agar (15). T6SS activity will lead to the formation of macroscopically visible zones of dead cells called Dienes lines between strains (15). Our mouse isolate strain of P. mirabilis formed Dienes lines with the type strain for P. mirabilis (ATCC 29906) and a human isolate (ATCC 7002) (red arrows, Fig. 3A). The three strains therefore utilize T6SS against nonkin strains. However, during coculture in shaking liquid media, no reduction in viability of either strain was observed (Fig. 3B and C). To confirm that killing is indeed independent of T6SS, we used a P. mirabilis *tssM* mutant (see Fig. S3 in the supplemental material), which lacks an important structural component of the T6SS and is deficient in T6SS-mediated effector delivery (15, 50–52). This mutant was generated in the P. mirabilis strain BB2000, which was shown to carry only a single T6SS (15, 51). Wild-type (WT) BB2000 and the *tssM* mutant strain reduced the viability of E. coli to the same extent and with the same kinetics (Fig. 3D and E). The mechanism used by P. mirabilis to reduce the viability of E. coli is therefore independent of T6SS activity.

10.1128/mSphere.00321-21.3 FIG S3 Confirmation of T6SS-deficient strain BB2000 *tssM.* Genomic region of BB2000_0808 encoding TssM/IcmF was amplified by PCR. WT BB2000 showed an expected band size of 3.5 kb, whereas the signal for the *tssM* mutant with the inserted pUTmini-Tn5-Cm transposon inserted showed the expected band size of ∼7 kb. Download FIG S3, JPG file, 0.1 MB. Copyright © 2021 Kiani et al. 2021 Kiani et al. https://creativecommons.org/licenses/by/4.0/ This content is distributed under the terms of the Creative Commons Attribution 4.0 International license.

### P. mirabilis kills E. coli rapidly and without prior contact.

**FIG 4:**
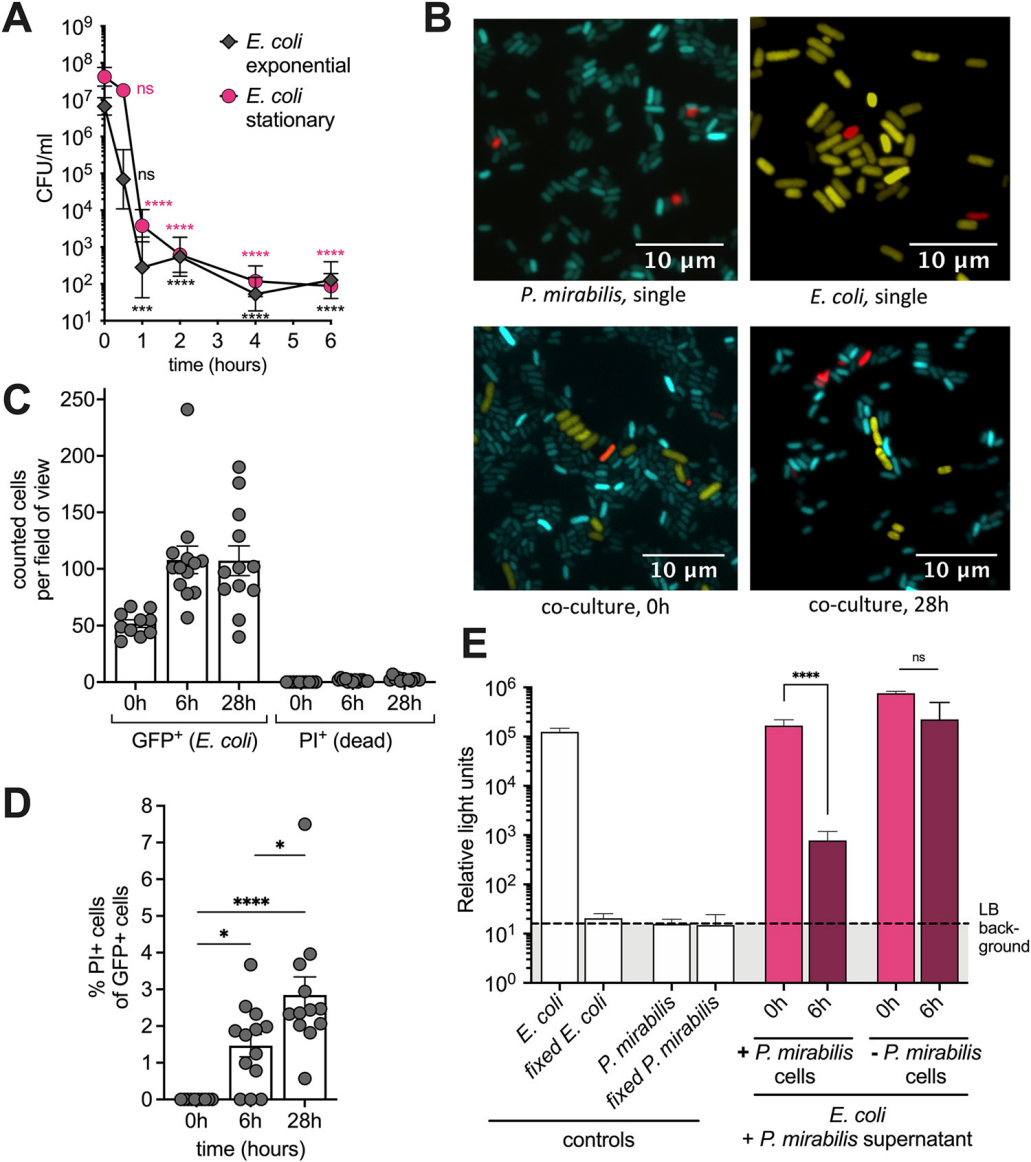
Killing is rapid and does not cause a loss of membrane integrity in target cells. (A) Exponential-phase or stationary-phase E. coli cells were added to a stationary P. mirabilis culture, and viability of E. coli was determined at indicated time points. A one-way ANOVA was performed comparing the 0-hour time point and remaining time points. (B) Representative images of YFP-expressing E. coli (yellow) and CFP-expressing P. mirabilis (cyan) stained for PI (red); top left to right: monoculture P. mirabilis, monoculture E. coli; bottom left to right, coculture at 0 hours and after 28 hours. (C and D) GFP-expressing E. coli was added to a stationary P. mirabilis culture (= “killing assay”), and culture was imaged 0 hours, 6 hours, or 28 hours after beginning of coculture. (C) Number of GFP-positive (E. coli) or propidium iodide (PI)-positive (P. mirabilis and E. coli) cells per field of view. (D) Percentage of GFP-positive (E. coli) cells that stain positive for PI. (E) Bioluminescent E. coli LuxAB in stationary phase was added to a stationary-phase P. mirabilis, and bioluminescence was measured immediately and after 6 hours. Controls: single-culture E. coli LuxAB and single-culture P. mirabilis, untreated or fixed in formalin. All data represent mean ± SEM of at least three biological replicates. For B, an ordinary one-way ANOVA test was used; and for C and D, Tukey’s multiple-comparison test was used. *, *P* < 0.05; ***, *P* < 0.001; ****, *P* < 0.0001; ns, not significant.

P. mirabilis does not kill E. coli when the coculture is actively growing in exponential phase. Some contact-dependent killing mechanisms require receptors on recipient surfaces for effector uptake (20, 53), which might not be expressed by E. coli during exponential growth phase (54). However, we found that regardless of the growth phase, E. coli rapidly lost viability when the cells were added to a stationary-phase culture of P. mirabilis. After 1 h of coincubation, we recovered about 13,000- and 23,000-fold reduced numbers of stationary-phase E. coli and exponential-phase E. coli, respectively (Fig. 4A). P. mirabilis therefore kills E. coli irrespective of the growth phase of E. coli. It also shows that P. mirabilis has the ability to kill E. coli without prior contact with E. coli during exponential phase.

### E. coli cells maintain cell shape and integrity despite the loss of viability and metabolic activity.

To gain a better understanding of the potential killing mechanism used by P. mirabilis, we used fluorescence microscopy. We imaged a coculture of cyan fluorescent protein (CFP)-expressing P. mirabilis and yellow fluorescent protein (YFP)-expressing E. coli and used propidium iodide (PI) to test for the membrane integrity of cells. After 28 h of coincubation, we did not observe changes in the number of YFP-expressing cells or their cellular morphology or an increase of PI-positive cells compared with single cultures (Fig. 4B). We also quantified our findings using GFP-expressing E. coli. The number of E. coli cells that we observed in each field of view by fluorescence microscopy was not reduced (Fig. 4C). According to CFU counts (Fig. 1H and 4A), at 6 h of coculture, almost all E. coli cells in each field of view should be nonviable. However, we observed very little PI uptake (1.5%) of GFP-positive (E. coli) cells (Fig. 4C and D). After 28 h of coculture, the percentage of PI-positive and GFP-positive cells increased only slightly to 2.9% (Fig. 4C and D) and the cellular morphology of GFP-positive cells remained unchanged (see Fig. S4A to D in the supplemental material). We next investigated if E. coli cells were still metabolically active using bacterial bioluminescence. When cells stop producing ATP, bioluminescence is rapidly lost. When bioluminescent E. coli cells were added to a stationary-phase culture of P. mirabilis, we observed a significant reduction in bioluminescence (Fig. 4E). No significant reduction in bioluminescence occurred when E. coli was grown for 6 h in stationary-phase P. mirabilis supernatant in the absence of P. mirabilis cells (Fig. 4E). Finally, we analyzed whether cells form aggregates in liquid culture. They might serve as a scaffold to allow for the prolonged cell contact often required for contact-dependent killing mechanisms. To preserve loose cell associations, samples were imaged directly as wet mounts. We occasionally observed an aggregate of P. mirabilis cells, but it was not associated with increased numbers of E. coli (Fig. S4E). Most cells at each time point were single cells (Fig. S4E). Nevertheless, E. coli cell viability decreased (Fig. S4F). Taken together, these data suggest that P. mirabilis compromises the metabolic activity of E. coli, but the mechanism does not result in a compromised cellular envelope in target cells.

10.1128/mSphere.00321-21.4 FIG S4 P. mirabilis does not form aggregates with E. coli in liquid culture and does not cause membrane integrity loss in target cells. (A to D) Representative images of P. mirabilis (unstained, brightfield) and E. coli (GFP-positive) stained with propidium iodide (red) (A) at the start of coculture (0 hour). Six hours (B) and 28 h (C) after the start of coculture and treated with 95% ethanol (EtOH) (D). (E) “Killing assay” of P. mirabilis (red) and E. coli (green). Three representative pictures of a wet mount at 0 h, 3 h, 6 h, and 28 h of coculture. Magnification of aggregates at 6 h and 28 h feature lower exposure to show individual cells in the aggregate. (F) Viability of P. mirabilis and E. coli at the time points depicted in E. Download FIG S4, JPG file, 1.0 MB. Copyright © 2021 Kiani et al. 2021 Kiani et al. https://creativecommons.org/licenses/by/4.0/ This content is distributed under the terms of the Creative Commons Attribution 4.0 International license.

### Activity of the killing system is differentially regulated in stationary phase.

**FIG 5:**
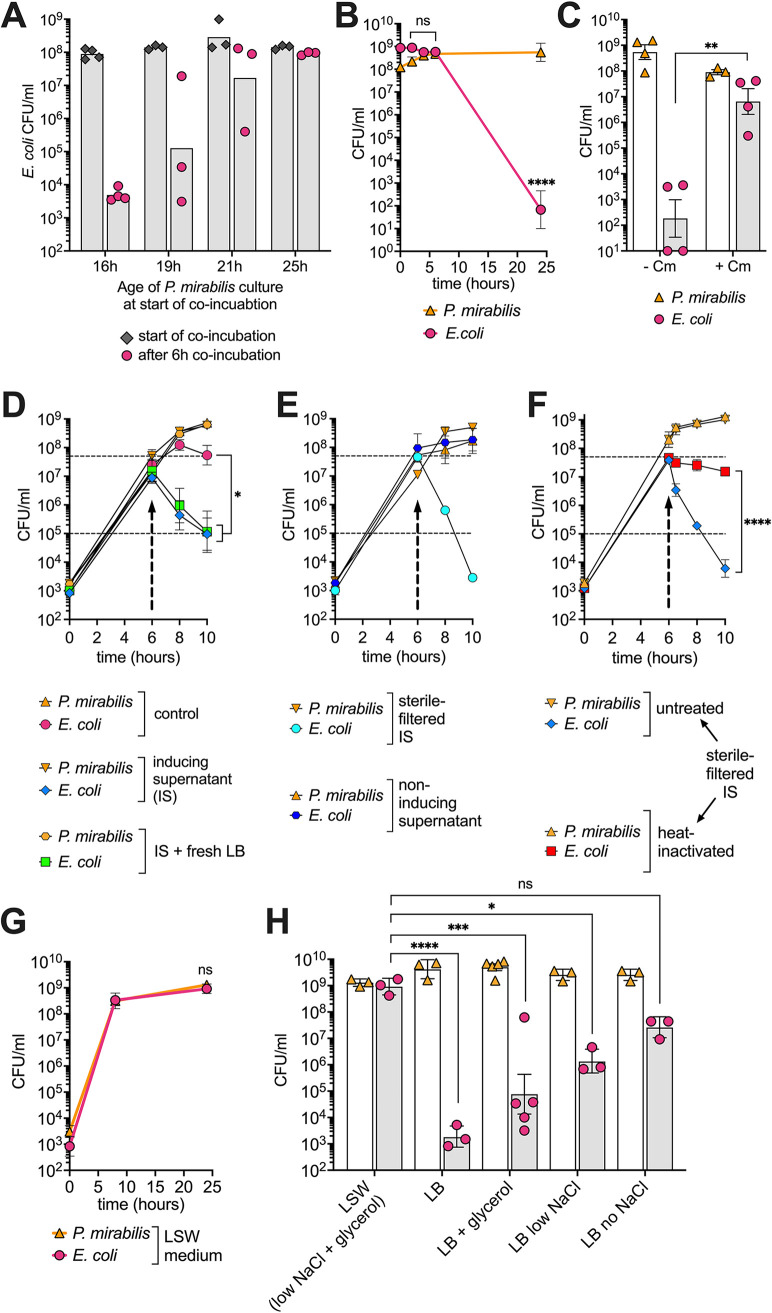
Killing requires new protein synthesis and is regulated by a component in the culture supernatant and osmolarity. (A) P. mirabilis was grown in single culture for 16, 19, 21, or 25 hours before stationary-phase E. coli cells were added. Viability of E. coli was assessed immediately or 6 hours after start of coincubation. (B) E. coli was grown in single culture for 16 hours before stationary-phase P. mirabilis cells were added. (C) E. coli was grown in single culture for 16 hours before stationary-phase P. mirabilis cells and chloramphenicol (15 μg/ml) were added. Histogram bar represents viability 24 hours after the beginning of coculture. (D to F) Killing induction assays. All data represent mean ± SEM of at least three biological replicates. For C and F, a Welch’s *t* test was performed. For D, a one-way ANOVA was performed comparing the viability of E. coli at the time of supernatant exchange and remaining time points. Upward arrow marks the time of supernatant exchange. The medium of a coculture of P. mirabilis and E. coli grown for 6 hours (indicated by arrows) was replaced with the growing culture’s own supernatant (control) (D). Supernatant of 22-hour-old culture of P. mirabilis (inducing supernatant [IS]). A 1:1 mixture of fresh LB and inducing supernatant (IS + fresh LB). (E) Sterile-filtered supernatant of 22-hour-old culture of P. mirabilis (sterile filtered IS). Supernatant of 22-hour-old culture of E. coli (noninducing supernatant). (F) Sterile-filtered supernatant of 22-hour-old culture of P. mirabilis (sterile filtered IS) either not treated (untreated) or boiled for 15 min (heat inactivated). (G) Shaking liquid growth curve of mouse P. mirabilis and E. coli in LSW (low NaCl, glycerol) broth. A Welch’s *t* test was performed between the 8-hour time point and the 24-hour time point comparing viability of E. coli. Ns, not significant. (H) Viability of E. coli and P. mirabilis after 24 hours of coculture in different media. A one-way ANOVA was performed comparing the viability of E. coli in different media to viability in LSW. *, *P* < 0.05; **, *P* < 0.01; ***, *P* < 0.001; ****, *P* < 0.0001; ns, not significant.

P. mirabilis rapidly reduces the viability of E. coli in a stationary-phase coculture (Fig. 4A). Interestingly, the number of remaining viable E. coli cells remains largely unchanged between 20 and 30 hours postinoculation (Fig. 1B). These remaining E. coli cells did not acquire resistance, as they were killed when isolated, grown, and exposed again to P. mirabilis (see Fig. S5A in the supplemental material). One possible explanation for the sustained viability of E. coli cells is that P. mirabilis downregulates expression of the killing system. Indeed, the capability of P. mirabilis to reduce the viability of added E. coli gradually diminished during stationary phase (Fig. 5A). When P. mirabilis cells were grown for 25 hours, they lost the ability to kill E. coli cells (Fig. 5A). At this time point, a culture of P. mirabilis also did not reduce the viability of *S.* Typhimurium (Fig. S5B). Taken together, these data suggest that as a P. mirabilis culture ages, the cells remain viable but lose the ability to kill E. coli.

10.1128/mSphere.00321-21.5 FIG S5 P. mirabilis in the later stage of stationary phase does not kill other *Enterobacteriaceae*. (A) E. coli that survived coculture with P. mirabilis for 24 h (passage 1) were subjected to a second coculture with P. mirabilis (passage 2), and surviving E. coli were again exposed to P. mirabilis (passage 3). Data represent three separate passaging experiments (*n* = 3). (B) P. mirabilis was grown in a single culture for 25 hours before *S*. Typhimurium cells were added. Viability of *S*. Typhimurium was assessed at the beginning of coculture and 6 hours after the start of coculture. Download FIG S5, JPG file, 0.2 MB. Copyright © 2021 Kiani et al. 2021 Kiani et al. https://creativecommons.org/licenses/by/4.0/ This content is distributed under the terms of the Creative Commons Attribution 4.0 International license.

The viability of E. coli cells was quickly reduced when placed in a stationary-phase P. mirabilis culture (Fig. 1H, Fig. 4A). However, when the inoculation was reversed and P. mirabilis cells were placed in a stationary culture of E. coli, killing was delayed. In this reversed setting, E. coli cells were alive 6 h postincubation with P. mirabilis cells (Fig. 5B). This finding prompted us to test whether new protein synthesis is required for killing. We used chloramphenicol to inhibit new protein synthesis. A chloramphenicol-resistant strain of E. coli was grown for 16 h, before 15 μg/ml of chloramphenicol and the chloramphenicol-sensitive P. mirabilis were added. This concentration of chloramphenicol is above the MIC (7.8 μg/ml) but does not interfere with P. mirabilis viability (Fig. 5C). The addition of chloramphenicol resulted in a significant (about 40,000-fold) rescue in the viability of E. coli compared with the control group without chloramphenicol (Fig. 5C), indicating that new protein synthesis is required for killing.

### Component(s) of the P. mirabilis supernatant regulate expression of the killing system.

P. mirabilis cells need to reach a high cell density before killing is observed (Fig. 1B and Fig. 5B). This finding might indicate that the behavior is regulated by quorum sensing (QS). QS is a form of bacterial cell-to-cell communication that involves the production, release, and response to extracellular molecules that collectively alter bacterial behavior (55). P. mirabilis cells might secrete QS molecules that accumulate in the supernatant as P. mirabilis culture density increases. These molecules might regulate expression, posttranslational modification, or use of the killing proteins. To test this hypothesis, we grew P. mirabilis and E. coli in a coculture to mid-exponential phase (6 hours). At this point, we replaced the growing culture supernatant with the supernatant of a stationary-phase (22 hour) culture of P. mirabilis, which could harbor putative QS molecules. This change indeed resulted in a loss of viability of E. coli in the coculture. Compared with the control group, we observed a highly significant 467-fold reduction in the viability of E. coli cells 4 hours after the supernatant exchange (Fig. 5D, blue line versus pink line). Nutrient deprivation and starvation were not responsible for the observed phenotype, as killing was also induced to the same level with a 50:50 mixture of P. mirabilis stationary-culture supernatant and fresh LB (Fig. 5D, green line). E. coli supernatant (noninducing) did not result in a loss of viability of E. coli (Fig. 5E). The sterile-filtered supernatant of P. mirabilis resulted in the same, if not even greater, viability loss of E. coli as nonsterile-filtered supernatant (Fig. 5D and E). We noticed that the proposed signaling molecule in the supernatant seems to be heat labile, as boiling of the 22-hour P. mirabilis supernatant was able to partially rescue E. coli viability (Fig. 5F, red line). Although P. mirabilis supernatant alone was not sufficient for killing E. coli (Fig. 2A), there seems to be a heat labile component within the supernatant that accumulates during the transition to stationary phase that regulates the P. mirabilis killing mechanism.

### The killing system is regulated via osmolarity.

The killing system also seems to be regulated by additional environmental factors. We discovered one such factor when we used the common P. mirabilis medium L swarm minus (LSW) (56) that inhibits swarming. In a coculture in LSW broth, P. mirabilis failed to kill E. coli (Fig. 5G). LSW and LB broth differ not only in their NaCl concentrations but also in glycerol as an additional carbon source in LSW. We therefore investigated the contribution of the individual components. In LB media supplemented with glycerol alone, E. coli was killed to almost the same extent as in LB. However, in LB with reduced NaCl concentration (0.4 g/liter), we observed a significant increase in the viability of E. coli cells. Complete omittance of NaCl from LB resulted in an even greater survival of E. coli cells. However, there was a trend of lower survival in LB without NaCl compared to LSW (Fig. 5H), suggesting that both low NaCl concentration and the presence of glycerol act to inhibit the expression and/or use of the killing system.

## DISCUSSION

### Novelty of the killing system.

Although a number of bacterial competition systems have been discovered, our understanding of bacterial warfare is still limited (13). The majority of contact-dependent killing mechanisms have been discovered and studied over just the past 15 years and a vast majority of organisms could harbor potentially novel mechanisms of competition (13). We initially observed what was apparently competition between a commensal P. mirabilis strain and a commensal E. coli in neonatal mice. During studies *in vitro*, we replicated the *in vivo* observation and showed that P. mirabilis killed competing E. coli. We have discovered that P. mirabilis utilizes a contact-dependent system to kill prey species (Fig. 2A and D). The known contact-dependent arsenal of weapons used by Gram-negative bacterial species against other species include T3SS, T4SS, T6SS, CDI (T5SS), Cdz, and MccPDI (13, 25). The inter-species killing system we describe is novel for P. mirabilis, as it is independent of T6SS (Fig. 3E). The system might also represent a novel system for bacteria in general, as the characteristics significantly differ from known contact-dependent mechanisms. We outline the differences between the P. mirabilis system and contact-dependent competition systems described in the literature in the following sections. However, killing could still be mediated through a not-yet-described mechanism or effector of one of these contact-dependent systems.

P. mirabilis encodes for a T3SS in its genome (57). T3SSs are widely used by bacteria to communicate with organisms belonging to other kingdoms, including protists, fungi, plants, and animals (58). While some bacteria use T3SS to kill eukaryotic yeast cells (59, 60), the current literature does not provide evidence for the use of T3SS as an interbacterial competition system. Alternatively, the T4SS has been described as an interspecies killing system for *Stenotrophomonas* and *Bartonella* sp., but it was effective only on solid media (61, 62).

The T6SS is widely used by Gram-negative species as an intra- and interbacterial killing mechanism (5, 44, 47, 51, 63–67) and represents the only contact-dependent killing mechanism described for P. mirabilis (57). However, the activity of P. mirabilis T6SS has only been shown against nonkin clonemates and not against other species (46, 68). As our study demonstrates, a mutant strain of P. mirabilis unable to produce a functional T6SS was able to kill E. coli (Fig. 3E). P. mirabilis therefore utilizes a mechanism that differs from the canonical T6SS.

Contact-dependent growth inhibition (CDI) depends on a two-partner secretion system and was first identified in E. coli liquid cultures (CdiA/CdiB) (42). The CDI system arrests growth in the target species but does not reduce viability. Moreover, the attacking strain has to be in exponential phase to deliver a toxic protein to the target cell. When the attacking strain is in stationary phase, as we see for P. mirabilis, it does not inhibit the target strain (42). Contrary to our data showing that a series of *Enterobacteriaceae* species are killed by P. mirabilis (Fig. 1), the effects of E. coli CDI do not expand to nonrelated species (20, 69, 70). Furthermore, CDI has been described to be predominantly active on solid media and not in shaking liquid culture (19, 22). One study on P. aeruginosa CDI showed some activity in liquid media. However, inhibition of susceptible strains is significantly reduced compared with solid surface conditions (43). It seems unlikely that P. mirabilis uses a CDI-like system against E. coli, as (i) killing of E. coli did not occur in exponential phase, (ii) distantly related species are killed, and (iii) contact-dependent killing occurred both on a solid surface and in shaking liquid media.

Another mechanism used by Gram-negative bacteria is contact-dependent inhibition by glycine zipper proteins (Cdz) (24). Caulobacter crescentus utilizes Cdz against susceptible C. crescentus strains and a limited number of species in the *Caulobacteraceae* family (53). The catalytic bacteriocin-like proteins CdzC and CdzD transfer through a receptor, presumably PerA (53), into the recipient cells and cause rapid depolarization of the cell membrane. An active Cdz system caused the vast majority (>95%) of target cells to lose membrane integrity, round up, and stain positive for propidium iodide (24). We did not observe a considerable uptake of PI or any changes in E. coli cellular morphology (Fig. 4B and C). Thus far, only the effector proteins CdzC and CdzD have been described for the Cdz system. P. mirabilis might use other effector proteins that differ in their mechanism of action against target bacteria. However, this is unlikely, as the authors of the study did not find homologs of the Cdz system in Proteus spp. (24).

Microcin proximity-dependent growth inhibition (MccPDI), identified in E. coli (25), occurs in shaking liquid media and requires direct contact or proximity between cells. However, killing or inhibition by the attacking strain occurs in late-exponential phase. Moreover, killing seems to be limited to different E. coli isolates (71). Finally, MccPDI requires the outer membrane protein OmpF on the target cells (72). Deletion of *ompF* rendered strains resistant, and expression of compatible OmpF rendered Salmonella enterica and Yersinia enterocolitica susceptible to E. coli MccPDI (72). However, P. mirabilis also killed an OmpF-deficient strain of E. coli (data not shown). Thus, the mechanism used by P. mirabilis to inhibit E. coli seems to be independent of MccPDI.

### Regulation of the killing system.

P. mirabilis reduced the viability of target species shortly after entering stationary phase. Presumably, the expression of the killing system itself or its regulator is upregulated upon reaching a concentration of above 10^9^ CFU/ml (Fig. 1). Interestingly, we found that the supernatant of P. mirabilis in stationary phase harbors component(s) that might function in a regulatory circuit controlling the activity of the killing system. Stationary-phase P. mirabilis culture supernatant was sufficient to induce killing by P. mirabilis in a coculture still in exponential phase. The component(s) in the supernatant might be signaling molecules in a quorum sensing (QS) regulatory network. As bacteria grow, they release QS molecules into their extracellular environment. Upon accumulation of these extracellular molecules, bacteria collectively alter many of their physiological behaviors such as competence, biofilm formation, and secretion systems (55, 73). Gram-negative microbes such as V. cholerae, P. aeruginosa, E. coli, and Serratia liquefaciens regulate their secretion systems, especially those known to be involved in bacterial killing, through QS (74). Many species of Pseudomonas and *Vibrio* regulate their killing mechanisms through QS molecules (64, 75–80). Gram-negative species have a plethora of mechanisms to synthesize QS molecules that include the production of homoserine lactones (HSLs) by LuxI/RpaI and autoinducer-2 (AI-2) by LuxS genes (55). P. mirabilis (strain BB2000) is known to harbor two genes involved in QS. The first is LuxS, encoding for AI-2 molecules, and the second is a receptor, RbsA, a homolog of LuxQ in *Vibrio* species (81). However, very few targets for QS systems in P. mirabilis have been identified. LuxS was reported to initiate swarming of P. mirabilis *in vitro*. A second report found a role for exogenously added HSLs in biofilm formation in P. mirabilis (82). However, there are no homologs of any genes encoding for HSLs in the genome of P. mirabilis. Nonetheless, these reports suggest a role for AI-2s and HSLs in regulating QS pathways in P. mirabilis. AI-2s or HSLs could therefore potentially be involved in the regulation of the P. mirabilis killing mechanism. The killing system also appears to be regulated by environmental factors, such as osmolarity and carbon source (Fig. 5H). Further studies are required to determine the nature and the mechanism by which putative signaling molecules and environmental factors govern the killing system used by P. mirabilis.

### Biological relevance.

Our study focuses on the *in vitro* characterization of how P. mirabilis reduces the viability of competitor species. However, it was informed by an *in vivo* observation in the mouse model with mouse-adapted gut commensal species. Our *in vitro* findings indicate that the system is active when P. mirabilis reaches a high cell density. *Enterobacteriaceae* levels are low in adult mice (Fig. S2B) and humans. However, in infants, *Enterobacteriaceae* species like P. mirabilis and E. coli initially dominate the microbiota and reach densities similar to that of stationary-phase growth *in vitro* (10^9^ CFU/g of fecal matter) (83, 84). It thus seems probable that P. mirabilis expresses the contact-dependent killing system in the infant gut and kills the competitor E. coli. However, we cannot exclude that what we observed in mouse pups is mediated or exacerbated by other members of the microbiota and not solely a result of direct *Enterobacteriaceae* competition alone. Therefore, future studies are needed to determine if and under which conditions P. mirabilis uses this killing system in the gut. P. mirabilis and E. coli are also well-known causes of urinary tract infections of humans. P. mirabilis is a concern for catheter-associated urinary tract infections (CAUTIs) and is frequently found in the presence of other microbes, including E. coli (85, 86). Whether P. mirabilis employs its putative interspecies competition system also in mixed biofilms will be an interesting question to address in future studies.

### Concluding remarks.

We report that P. mirabilis is able to reduce the viability of a variety of *Enterobacteriaceae* species in a contact-dependent manner. The reduction of viability is independent of the T6SS, which is the only contact-dependent killing system described for P. mirabilis. To our knowledge, the mechanism is novel for P. mirabilis. The present study is a first description and characterization of the observed P. mirabilis-mediated interbacterial killing phenotype. The identities of the genetic components required for the expression of the system are still unknown and are the focus of ongoing studies. Similarly, the current study indicates possible regulatory mechanisms for the system, which are being investigated. As four different P. mirabilis isolates reduced the viability of E. coli, it might be a capability that is widespread among P. mirabilis strains. Frequently, competition systems are also not restricted to one species but can be found in multiple species, as shown for T6SS, T4SS, CDI, and Cdz. The competition system we identified in P. mirabilis might therefore also be present in other species.

## MATERIALS AND METHODS

### Mouse and bacterial strains.

The female C57BL/6 SPF mouse used for breeding originated from a rederivation with Envigo CD-1 mice and thus harbors CD-1 microbiota. The male C57BL/6 SPF mouse used for breeding was obtained from Taconic laboratories. Both the Escherichia coli and Proteus mirabilis isolated from these mice are natural colonizers of the gastrointestinal tract of these animals (Table 1). The dam and the sire were allowed to mate, and both were kept in the same cage before the pups were weaned at approximately 21 days of age. Fresh fecal samples were obtained at weaning from all seven pups and homogenized in 1 ml phosphate-buffered saline (PBS), and 100 μl of the homogenate was plated onto MacConkey agar plates. Determination of colonization status was qualitative; only translucent (P. mirabilis) and no pink (E. coli) colonies were recovered. In order to determine the colonization status of an additional 78 mice, fresh fecal samples were collected, weighed, and homogenized in 1 ml PBS, and 100 μl of each homogenate was plated onto MacConkey agar plates. Translucent (P. mirabilis) and E. coli (pink) colonies were counted, and colonization for each species was expressed as CFU/mg feces. Mice colonized with either E. coli (C575BL/6 mice originating from Envigo CD-1 rederivation) or P. mirabilis (C57BL/6 mice originating from breeding experiment) were cohoused for 276 days (2 mice) or 581 days (4 mice). Mouse breeding and isolation of bacterial strains were performed at the University of California, Irvine. All animal experiments were reviewed and approved by the Institutional Animal Care and Use Committee at the University of California, Irvine.

**TABLE 1:** Bacterial strains

Designation	Strain ID^*a*^	Genotype	Host or other information	Source or reference
Mouse Escherichia coli (JB2)	BL27	WT	Mouse	41
Mouse E. coli GFP	BL143	BL27 + pJC43		This study
Mouse E. coli YFP	BL283	BL27+ pMRE-133		This study
Mouse E. coli Cm^r^	BL30	BL27+ pACYΩ		This study
Mouse Proteus mirabilis	BL95	WT	Mouse	This study
Mouse P. mirabilis, Carb^r^	BL125	BL95 + pHP45Ω		This study
Mouse P. mirabilis CFP	BL285	BL95 + pUCP30T-CFP		This study
Mouse P. mirabilis Crimson	BL225	BL95 + pUCT30-E2		This study
P. mirabilis ATCC 7002	BL139	WT	Human	ATCC
P. mirabilis ATCC 7002, Cm^r^	BL149	BL139 + pACYΩ		This study
P. mirabilis ATCC 29906 (CDC PR 14)	BL141	WT	Type strain	ATCC
P. mirabilis ATCC 29906, Cm^r^	BL150	BL141 + pACYΩ		This study
P. mirabilis BB2000	BL279	WT	Human	68
P. mirabilis BB2000 T6SS mutant	BL280	Δ*tssM*		15
Salmonella enterica serovar Typhimurium IR715, Carb^r^	BL2	ATCC 14028, spontaneous Nal^r^ derivative + pHP45Ω		41
Listeria monocytogenes 10403S	BL220	WT, Strep^r^		87
Candida albicans ATCC 90028	BL231	WT		ATCC
Klebsiella pneumoniae KPPR1	BL221	WT		ATCC
Klebsiella pneumoniae KPPR1 (capsule deficient)	BL268	Strain MJM2462 *wza::*Tn*kan*		Acadia Kocher and Mark Mandel
Citrobacter rodentium DBS 100 (ATCC 51459)	BL222	WT		ATCC
E. coli LuxAB	BL248	E. coli SURE+ p7INT-recA_luxAB		88

a ID, identifier.

### Media and growth conditions.

P. mirabilis, E. coli, C. rodentium, *S.* Typhimurium, and K. pneumoniae were routinely cultured in lysogeny broth (LB). C. albicans was cultured in yeast extract-peptone-dextrose (YPD) broth. L. monocytogenes was cultured in brain heart infusion (BHI) broth. Cultures were grown shaking in a 20-mm-diameter culture tube at 200 rpm for 16 hours in 5 ml of medium unless otherwise indicated. P. mirabilis BL125 was selected on MacConkey agar plates supplemented with carbenicillin (100 mg/liter). P. mirabilis strains BL149 and BL150 were selected on MacConkey agar supplemented with chloramphenicol (30 mg/liter). E. coli strains BL143 and BL30 were selected on MacConkey agar plates supplemented with kanamycin (50 mg/liter) and chloramphenicol (30 mg/liter), respectively. *S.* Typhimurium was selected on LB agar supplemented with carbenicillin (100 mg/liter). Wild-type and capsule-deficient K. pneumoniae cells were selected on MacConkey agar supplemented with carbenicillin (100 mg/liter) and kanamycin (50 mg/liter), respectively. L. monocytogenes was selected on BHI agar plates supplemented with streptomycin (100 mg/liter). P. mirabilis strains BL95, BL279, and BL280 were selected for on MacConkey agar plates with no antibiotics. C. albicans was selected for on Sabouraud agar supplemented with carbenicillin (100 mg/liter). For the LSW cocultures, BL95 was selected for on LB agar plates with tetracycline (20 mg/liter).

### Monoculture growth assays.

E. coli BL143 was grown in LB for 16 hours. The strain was centrifuged at 9,400 relative centrifugal force (rcf), resuspended, and diluted in sterile PBS. Twenty milliliters of LB was inoculated with 5 × 10^3^ CFU/ml of BL143. The culture was incubated shaking at 200 rpm at 37°C, and an aliquot (100 μl) was collected at 0, 2, 5, 8, 16, and 24 hours. This aliquot was resuspended in sterile PBS, serially diluted, and plated onto MacConkey agar.

### Coculture growth assays.

P. mirabilis BL95 and its respective antagonist were grown separately in LB for 16 hours. Each strain was centrifuged at 9,400 rcf, resuspended, and diluted in sterile PBS. Twenty milliliters of LB was inoculated with 5 × 10^3^ CFU/ml of P. mirabilis BL95 and antagonist. The coculture was incubated shaking at 200 rpm at 37°C, and an aliquot (100 μl) was collected at 0, 2, 5, 8, 10, 14, 16, 18, 20, 24, and 30 hours. This aliquot was resuspended in sterile PBS, serially diluted, and plated onto agar plates with antibiotics to select for P. mirabilis or the antagonist.

### Killing assay.

P. mirabilis and Gram-negative antagonist species were grown separately in 5 ml of LB for 16 hours shaking at 37°C. L. monocytogenes was grown in 5 ml of brain heart infusion (BHI) at 37°C, and C. albicans was grown in 5 ml of yeast extract-peptone dextrose (YPD) at 30°C in shaking liquid culture. A total of 5 × 10^8^ cells of the antagonist was centrifuged (9,400 rcf) and resuspended in 500 μl of sterile PBS. The cells were added to 4.5 ml of the P. mirabilis culture. This amount roughly corresponds to an attacking to target ratio of 10:1. The viability of the antagonists was measured by plating on their respective media supplemented with antibiotics (see section above).

### Supernatant assay.

P. mirabilis BL95 and E. coli BL143 cells were grown for 16 hours. The P. mirabilis culture was centrifuged at 3,220 rcf for 15 minutes, and the supernatant was sterile filtered using a 0.22-μm syringe filter. A total of 10^8^ E. coli cells were centrifuged at 9,400 rcf for 5 minutes and resuspended in PBS. E. coli cells were added to the sterile-filtered supernatant of P. mirabilis and incubated shaking at 200 rpm at 37°C. The viability of E. coli cells was measured 6 and 24 hours after the start of incubation in P. mirabilis supernatant.

### Split well assay.

In this assay, cultures were separated by membranes of different pore sizes in 6-well plates (VWR). Inserts with a membrane pore size of 0.4 μm (10769-192; VWR) or 8.0 μm (10769-196; VWR) were placed in wells of the plate, creating two chambers. A total of 2 ml of fresh LB was added to each chamber. Cultures (16 h old) of BL30 and P. mirabilis cells BL125 were centrifuged (9,400 rcf), resuspended, and diluted in sterile PBS. The upper and lower chamber were inoculated with 5 × 10^3^ cells of E. coli BL30 and P. mirabilis BL125, respectively. Plates were incubated shaking at 90 rpm at 37°C. The viability of the cells in each chamber was measured by plating the cells on medium selective for either P. mirabilis or E. coli.

### Solid surface assay.

P. mirabilis BL95, E. coli BL143, K. pneumoniae BL221, and S. enterica serovar Typhimurium BL2 cells were grown in shaking liquid culture for 16 hours. A total of 10 μl of each strain containing either 10^8^ (high density) or 10^3^ (low density) cells was mixed and immediately spotted either onto MacConkey agar or LB agar. The plates were incubated at 37°C (Fig. 2) or 30°C (Fig. S2) for 24 hours. After 24 hours, a 9-mm-diameter hole puncher was used to stab the center of the growing colony. The agar plug was placed in 2 ml of PBS, and cells were resuspended by gentle vortexing. Cells were serially diluted and plated onto medium selective for either strain.

### Formalin treatment.

P. mirabilis BL95 was grown for 16 hours, and 5 ml of the culture was centrifuged (9,400 rcf for 10 minutes). The supernatant was sterile filtered. The cells were resuspended in 1 ml of 10% formalin, incubated for 5 minutes at room temperature, and washed twice with 1 ml PBS to remove any residual formalin. After the final wash, cells were either resuspended in 5 ml LB or the original sterile-filtered P. mirabilis culture supernatant. E. coli was grown for 16 hours, and 5 × 10^8^ cells were added to each tube. The cultures were placed in a shaker at 37°C shaking at 200 rpm, and the viability of E. coli cells was measured by plating serial dilutions on selective media at 6 and 24 hours after the start of coculture.

### Chloramphenicol treatment.

P. mirabilis BL95 and E. coli BL30 cells were grown for 16 hours. A total of 5 × 10^8^ P. mirabilis cells were centrifuged (9,400 rcf for 5 minutes) and resuspended in sterile PBS. Five hundred microliters of P. mirabilis cells was placed into 4.5 ml of an E. coli culture grown for 16 hours. Chloramphenicol (15 μg/ml) was then added to the coculture. Samples were taken over a 24-hour period, serially diluted, and plated onto selective media to assess viability. E. coli and P. mirabilis were selected on MacConkey agar with chloramphenicol and MacConkey with no antibiotics, respectively.

### Fluorescence microscopy.

E. coli BL143 cells expressing green fluorescence protein (GFP) and P. mirabilis BL95 were inoculated in a killing assay (see respective section). At indicated time points, 100 μl of the coculture was centrifuged at room temperature for 10 minutes at 9,400 rcf. The pellet was resuspended in 1 ml of propidium iodide (PI) in sterile PBS (final concentration of 2 × 10^−3 ^mg/ml) and incubated for 15 minutes protected from light. Cells (10 μl) were spotted onto a 1 by 1-cm-wide, 2-mm-thick agarose pad (1.5% agarose in PBS) positioned on a microscope slide and incubated 10 minutes to dry. Once the sample was absorbed onto the agarose pad, a cover slip was added and sealed with nail polish. Control cells were treated with 95% EtOH before addition of PI. The cells were visualized at 60× in a Keyence inverted microscope with GFP (excitation, 470/40; emission, 525/50 nm) and Texas Red (excitation, 560/40; emission, 630/75 nm) filter sets. Ten representative images were taken per slide, averaging 100 to 300 GFP-positive cells per field. Each image was analyzed for the total number of GFP-positive and PI-positive cells.

E. coli cells expressing YFP (BL283) were grown for 16 hours with 0.5% arabinose. P. mirabilis cells expressing CFP (BL285) were grown for 16 hours with 1 mM isopropyl-β-d-thiogalactopyranoside (IPTG). A killing assay as described above was performed. Representative images of the assay were taken at the start of the assay (0 hour) and 28 hours later. A representative image of the single culture of each strain was taken 28 hours after inoculation. Images were taken with a Zeiss AxioImager Z2 upright microscope. The cells were visualized at 63×/1.40 oil DIC M27 magnification with CFP (excitation, 433; emission, 475 nm), YFP (excitation, 508; emission, 524 nm), and RFP (excitation, 590; emission, 612 nm) filter sets.

For fluorescence microscopy of liquid coculture, P. mirabilis BL225 and E. coli BL143 were grown for 16 h in 5 ml of LB + 1 mM IPTG and 5 ml of LB, respectively. A total of 5 × 10^8^ cells of E. coli were centrifuged (9,400 rcf), resuspended in 500 μl of sterile PBS, and added to 4.5 ml of the P. mirabilis culture. At the indicated time points, 20 μl of the coculture was removed with a wide bore tip and placed on a 22 by 50-mm coverslip. A second coverslip of 18 by 18 mm was placed on top, and the cells were visualized in a Keyence inverted microscope with GFP (excitation, 470/40; emission, 525/50 nm) and Texas Red (excitation, 560/40; emission, 630/75 nm) filter sets.

For fluorescence microscopy of killing on a solid surface, P. mirabilis BL225 and E. coli BL143 were grown for 16 h in 5 ml of LB + 1 mM IPTG and 5 ml of LB, respectively. A total of 10 μl of suspension containing either 1 × 10^3^ (low density) or 1 × 10^8^ (high density) of each strain was spotted on thin, 2-mm-thick LB (swarming) and MacConkey (nonswarming) agar plates. At the indicated time points, a piece of agar of 1 by 1 cm was excised with a scalpel, placed on a 22 by 50-mm coverslip facing down (placing the side containing the bacteria in contact with the coverslip), and imaged using a Keyence inverted microscope with GFP (excitation, 470/40; emission, 525/50 nm) and Texas Red (excitation, 560/40; emission, 630/75 nm) filter sets. For Movie S1, 67 images, taken with both GFP and Texas Red channels, were captured every 25 seconds for a total of 30 minutes. Images were then processed with the software ImageJ, and the movie sequence was created using the software iMovie.

### Luminescence assays.

Luminescent E. coli BL248, nonluminescent E. coli BL27, and P. mirabilis BL95 were grown for 16 hours. The P. mirabilis culture was centrifuged at 3,220 rcf for 15 minutes. A total of 4.5 ml of the supernatant was sterile filtered using a 0.2-μm syringe filter (28145-501; VWR). A total of 5 × 10^8^ cells of luminescent E. coli BL248 were centrifuged (5 minutes at 9,400 rcf), resuspended in 500 μl of sterile PBS, and inoculated either into the sterile filtered supernatant of P. mirabilis or 4.5 ml of P. mirabilis (killing assay). A total of 1 × 10^9^ E. coli BL248 and P. mirabilis were centrifuged (5 minutes at 9,400 rcf), resuspended in 500 μl of 10% formalin, and incubated 5 minutes. Formalin-treated cells were washed once with 500 μl of sterile PBS. A total of 100 μl (two technical replicates) of each condition was aliquoted into 96-well plates (655083; Greiner Bio-One) and exposed to decyl aldehyde (A0398589; Sigma) fumes. Luminescence was measured using a Biotek Synergy 2 instrument with the following settings: integration time, 0:01.00 (MM:SS.ss); filter set 1 emission, Hole Optics:Top Gain: AutoScale; read speed, Normal; and delay, 100 msec. The average between each technical replicate was designated one biological replicate.

### Killing induction assays.

To obtain supernatants for killing induction, a culture of P. mirabilis BL95 was grown for 22 hours. Cells were centrifuged at 3,220 rcf for 20 minutes, and supernatants were obtained by decanting. Supernatants were either used directly in assays or were sterile filtered before use. For the killing induction assay, E. coli cells (BL143) and P. mirabilis cells (BL95) were grown for 16 hours separately. A total of 10^10^ cells of each strain were centrifuged at 9,400 rcf for 5 minutes and resuspended in 500 μl of sterile PBS. A total of 5 × 10^3^ cells of each strain were inoculated in 5 ml of fresh LB and incubated shaking at 37°C and 200 rpm. The coculture was grown for 6 hours and centrifuged at 3,220 rcf for 15 minutes. The pellet was resuspended in 5 ml of different media, namely, its own supernatant, 22-hour supernatant of P. mirabilis (inducing supernatant), 2.5 ml of fresh LB and 2.5 ml of inducing supernatant, boiled inducing supernatant (95°C for 15 minutes), 22-hour supernatant of E. coli, or 22-hour sterile-filtered supernatant of P. mirabilis.

### Coculture assay in LSW.

P. mirabilis BL95 and E. coli BL143 were grown in LSW for 16 hours. Each strain was centrifuged at 9,400 rcf, resuspended, and diluted in sterile PBS. Twenty milliliters of LSW was inoculated with 5 × 10^3^ CFU/ml of each strain. The coculture was incubated shaking at 200 rpm at 37°C, and an aliquot (100 μl) was collected at 0, 8, and 24 hours. This aliquot was resuspended in sterile PBS, serially diluted, and plated on agar plates with antibiotics to select for P. mirabilis (LB + tetracycline) or E. coli (MacConkey + kanamycin). To investigate the individual components of LSW broth, the media in Table 2 were prepared, the pH adjusted to 7, and autoclaved.

**TABLE 2:** Medium composition

Medium	NaCl (g/liter)	Tryptone (g/liter)	Yeast extract (g/liter)	Glycerol (ml/liter)
LB	10	10	5	0
LSW	0.4	10	5	5
LB glycerol	10	10	5	5
LB low NaCl	0.4	10	5	0
LB no NaCl	0	10	5	0

### Swarming assays.

Ten microliters of 16-hour-old cultures of P. mirabilis strains (BL95, BL139, and BL141) was spotted onto freshly poured LB agar plates (2% agar). Plates were incubated at 37°C for 48 hours.

### Re-exposure assays.

E. coli BL143 and P. mirabilis BL95 were grown as described in “Coculture growth assays” (passage 1). CFUs were determined 24 h postinoculation. For the second passage, a colony from each strain was selected and placed in 20 ml LB broth. Bacteria were plated and enumerated 24 hours later. For the third passage, a colony from each strain was again placed in 20 ml LB broth, and CFUs were determined 24 h later.

### Confirmation of BB2000 *tssM* mutant.

The P. mirabilis BB2000 WT and *tssM* mutant were gifts from Karine Gibbs. The mutant in *tssM* was generated via an insertion of Tn5-Cm into the *tssM* locus of BB2000 (15). The lack of functionality of T6SS was confirmed previously in references 15 and 50–52. Strain identity and integration of Tn5-Cm were confirmed via PCR with the primers 5′-TTCAAACATTACGAGTTCGG-3′ and 5′-TTCAGGTAAACGGAATTTTGTG-3′ amplifying the *tssM* locus.

### Statistical analysis.

Ordinary one-way ANOVA followed by multiple comparisons and nonpaired Welch *t* tests were performed where indicated using GraphPad Prism version 9.0.0 for MacOS, GraphPad Software, San Diego, CA, USA.

### Data availability.

All relevant data are within the manuscript and its supplemental material.
