# Supplementary figures and images for: Proteus mirabilis Employs a Contact-Dependent Killing System against Competing Enterobacteriaceae
Source: mSphere. 2021 Jul 28;6(4):e00321-21. doi: 10.1128/mSphere.00321-21 (PMC8386478; doi:10.1128/mSphere.00321-21)

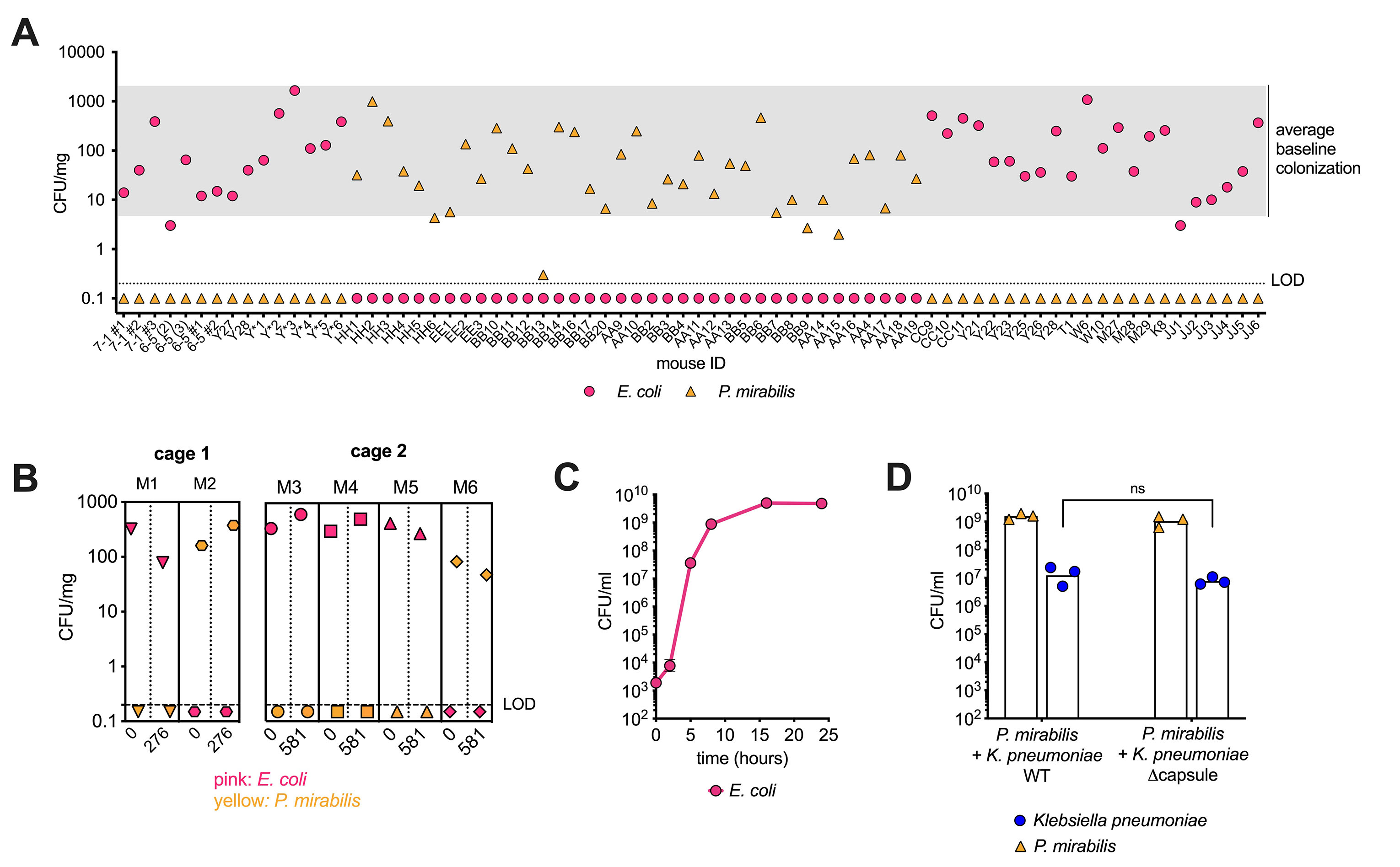

Supplement: FIG S1 [file msphere.00321-21-sf001.jpg]

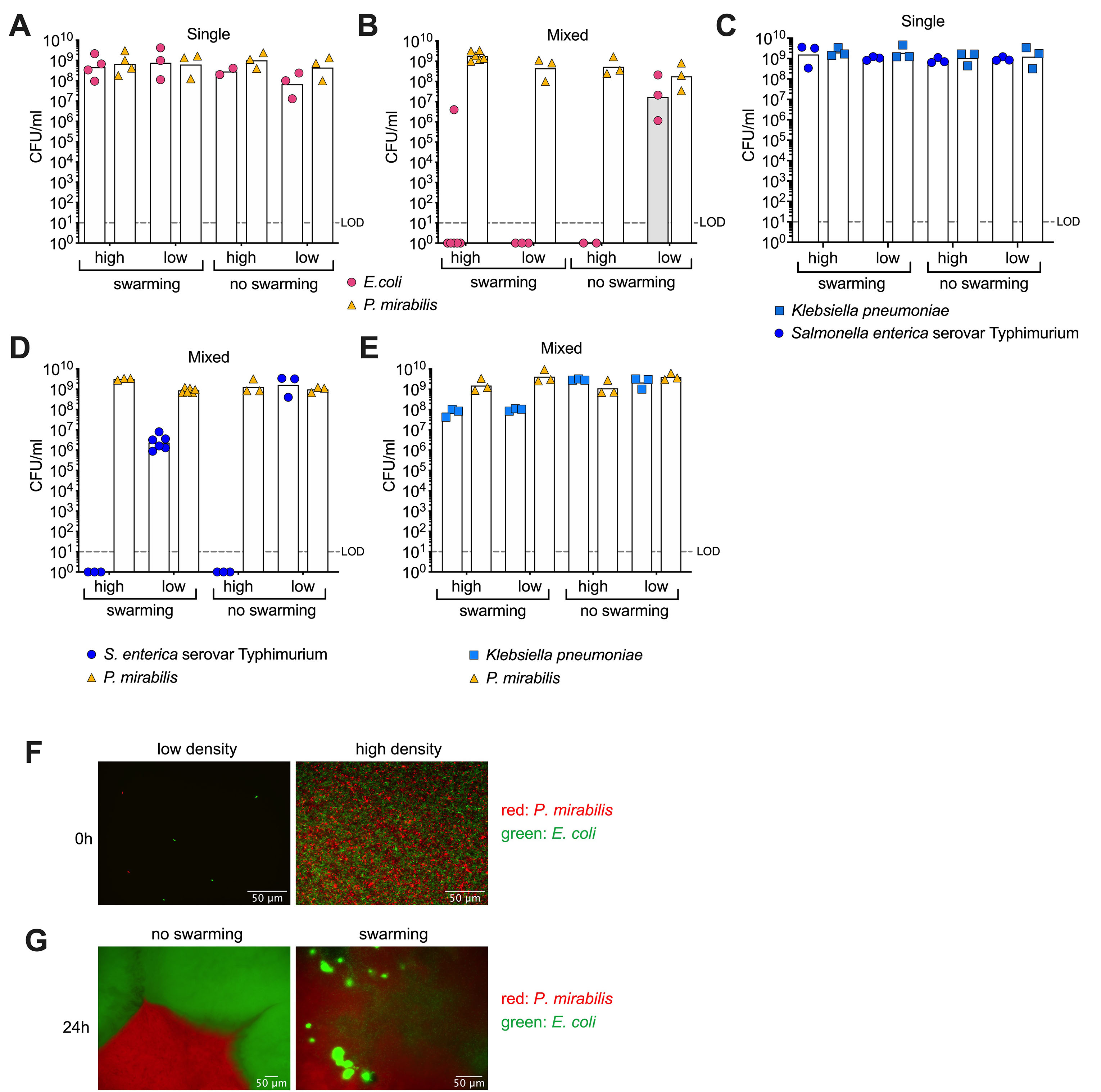

Supplement: FIG S2 [file msphere.00321-21-sf002.jpg]

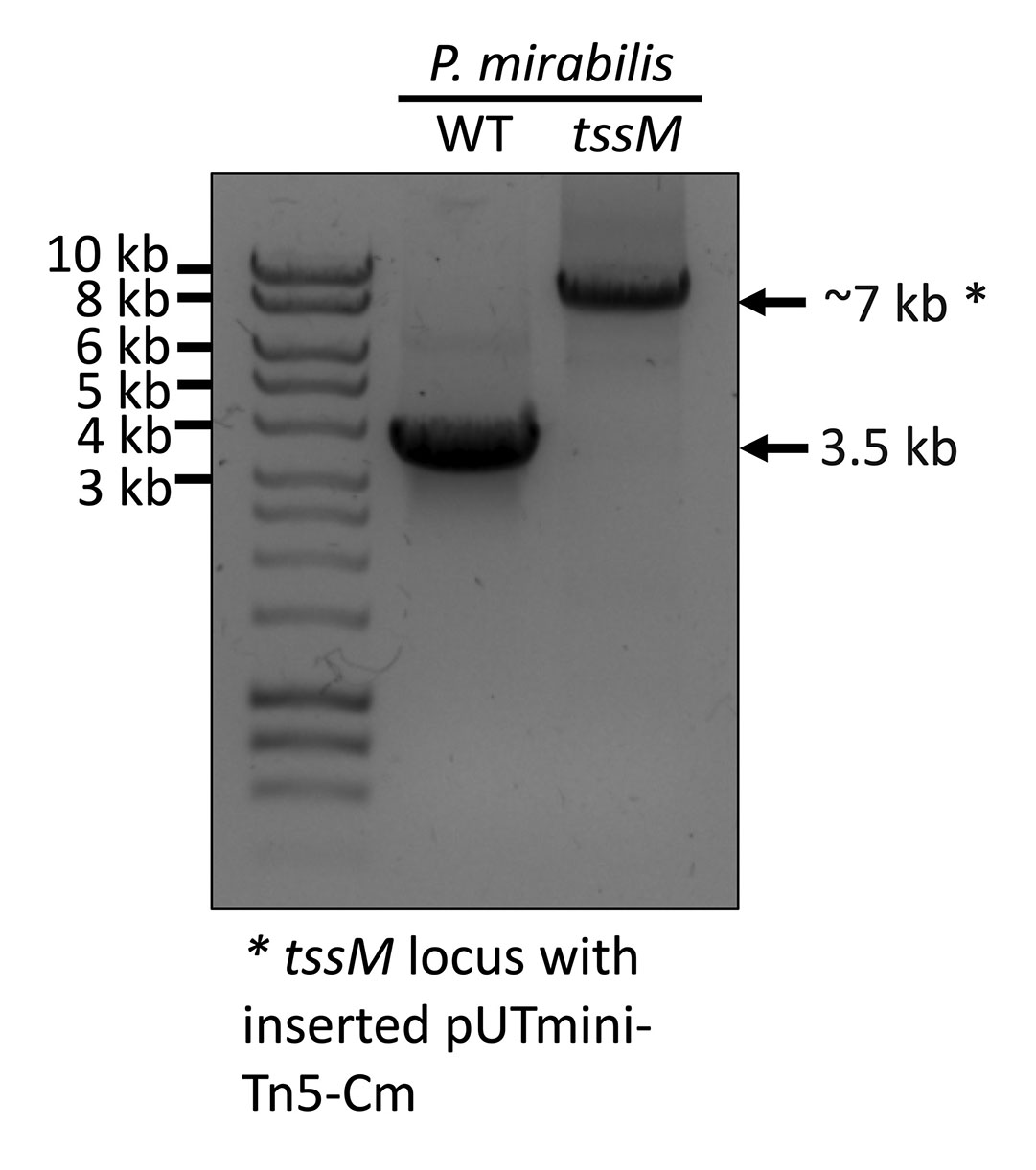

Supplement: FIG S3 [file msphere.00321-21-sf003.jpg]

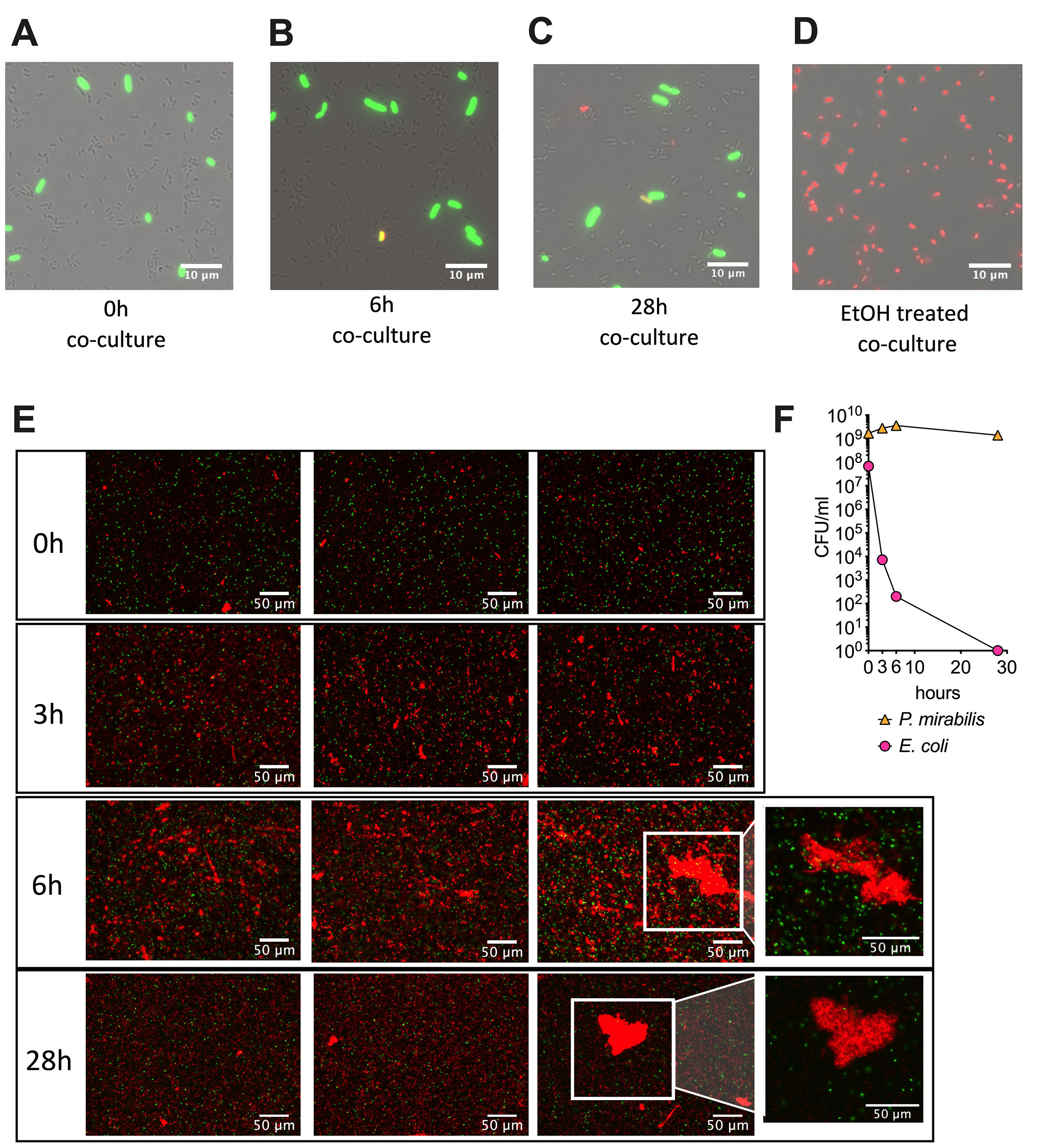

Supplement: FIG S4 [file msphere.00321-21-sf004.jpg]

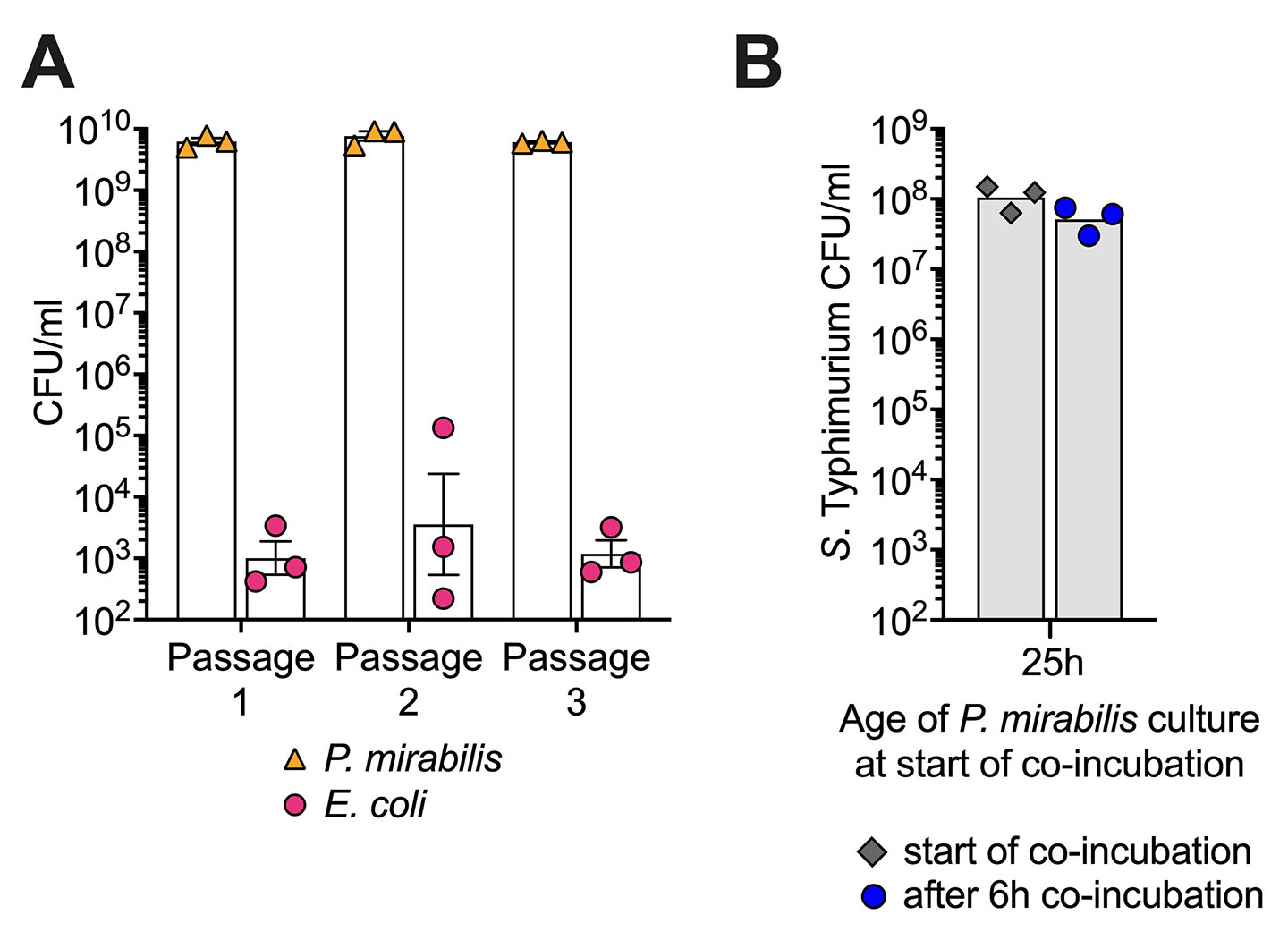

Supplement: FIG S5 [file msphere.00321-21-sf005.jpg]
